# Stain normalization gives greater generalizability than stain jittering in neural network training for the classification of coeliac disease in duodenal biopsy whole slide images

**DOI:** 10.1016/j.jpi.2023.100324

**Published:** 2023-07-19

**Authors:** B.A. Schreiber, J. Denholm, J.D. Gilbey, C.-B. Schönlieb, E.J. Soilleux

**Affiliations:** a Department of Pathology, University of Cambridge, Cambridge, UK; b Department of Applied Mathematics and Theoretical Physics, University of Cambridge, Cambridge, UK; c Lyzeum Ltd., Cambridge, UK

**Keywords:** Coeliac, Neural network, Stain normalization, Stain jittering, Supervised learning

## Abstract

Around 1% of the population of the UK and North America have a diagnosis of coeliac disease (CD), due to a damaging immune response to the small intestine. Assessing whether a patient has CD relies primarily on the examination of a duodenal biopsy, an unavoidably subjective process with poor inter-observer concordance. Wei et al. [11] developed a neural network-based method for diagnosing CD using a dataset of duodenal biopsy whole slide images (WSIs). As all training and validation data came from one source, there was no guarantee that their results would generalize to WSIs obtained from different scanners and laboratories. In this study, the effects of applying stain normalization and jittering to the training data were compared. We trained a deep neural network on 331 WSIs obtained with a Ventana scanner (WSIs; CD: n=190; normal: n=141) to classify presence of CD. In order to test the effects of stain processing when validating on WSIs scanned on varying scanners and from varying laboratories, the neural network was validated on 4 datasets: WSIs of slides scanned on a Ventana scanner (WSIs; CD: n=48; normal: n=35), WSIs of the same slides rescanned on a Hamamatsu scanner (WSIs; CD: n=48; normal: n=35), WSIs of the same slides rescanned on an Aperio scanner (WSIs; CD: n=48; normal: n=35), and WSIs of different slides scanned on an Aperio scanner (WSIs; CD: n=38; normal: n=37).

Without stain processing, the F1 scores of the neural network were 0.947, 0.619, 0.746_,_ and 0.727 when validating on the Ventana validation WSIs, Hamamatsu and Aperio rescans of the Ventana validation WSIs, and Aperio WSIs from a different source respectively. With stain normalization, the performance of the neural network improved significantly with respective F1 scores 0.982, 0.943, 0.903, and 0.847. Stain jittering resulted in a better performance than stain normalization when validating on data from the same source F1 score 1.000, but resulted in poorer performance than stain normalization when validating on WSIs from different scanners (F1 scores 0.939, 0.814_,_ and 0.747). This study shows the importance of stain processing, in particular stain normalization, when training machine learning models on duodenal biopsy WSIs to ensure generalizability between different scanners and laboratories.

## Introduction

Coeliac disease (CD) is an autoimmune disorder triggered by the consumption of gluten.^1^ While CD was once seen as a rare childhood disease, it is now recognized as a common condition,^2^ and around 1% of the population of the UK and North America have been diagnosed, with increasing prevalence in the UK.^3^^,^^4^ As many as 8 affected individuals out of 9 may remain undetected^5^ and manifestations are variable and non-specific, ranging from no symptoms, through anaemia to severe intestinal symptoms, with complications including bone thinning, infertility and, rarely, lymphoma and duodenal cancer.^6^ Since treatment is relatively straightforward, involving a lifelong gluten-free diet,^7^ accurate diagnosis is of critical importance.

While serological tests are often used as a screening tool for patients suffering from symptoms of CD, the gold-standard for assessing whether a patient has CD relies primarily on a small intestinal biopsy examination by a pathologist.^8^ This is an unavoidably subjective process with poor inter-observer concordance.^9^^,^^10^ Frequent false-negative and equivocal results are contributing factors to the average time from symptom onset to diagnosis being approximately 13 years.^7^ Accordingly, this study describes the first part of a strategy to develop a more sensitive, objective, reproducible, and clinically deployable diagnostic tool.

**Fig. 1:**
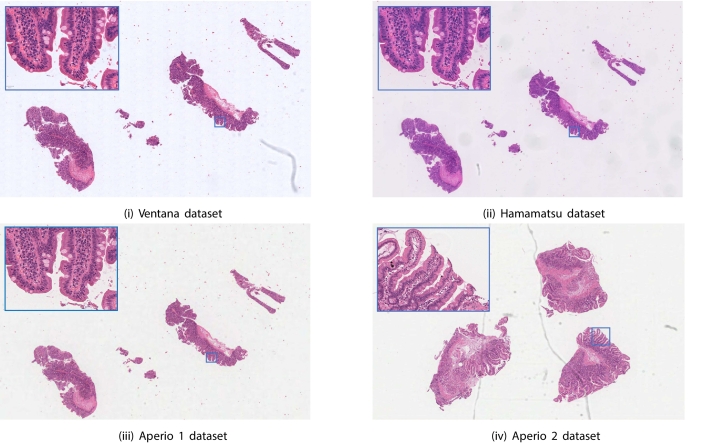
Examples from the 4 datasets. An example patch at ×20 magnification is shown in a blue square for each example. (i) An example from the Ventana validation dataset. Each WSI in the Ventana dataset contains 2–6 pieces of tissue. (ii, iii) The WSI in the Hamamatsu and Aperio 1 datasets, respectively, which are rescans of example (i). While the morphology of the biopsies remain the same, the stain absorption vectors and background colour do not. (iv) An example from the Aperio 2 dataset. The Aperio 2 dataset contains WSIs of different biopsies stained in a different laboratory. The stain absorption vectors and background colour are similar to (iii).

Recently, there have been a number of publications discussing the automation of this process using machine learning.^11^, ^12^, ^13^ For example, Wei et al.^11^ proposed a deep neural network for diagnosing CD in duodenal biopsies. Biopsies underwent laboratory processing, producing microscope slides that were visualized with hematoxylin and eosin (H&E) staining, causing the nuclei to appear blue and the cytoplasm and lamina propria pink.^14^ These slides were scanned with an Aperio whole-slide scanner to make a dataset of duodenal biopsy whole slide images (WSIs). A ResNet architecture^15^ was used to classify whether duodenal biopsies were negative or positive for CD, or showed signs of non-specific duodenitis. However, all experiments were conducted on WSIs obtained from the same source. Due to the black-box nature of neural networks, it is unclear whether their method would generalize to other sources. Ensuring generalizability between WSIs from different sources is essential for developing a trusted diagnostic tool, as the differences between data from different sources can be marked (see Fig. 1).

A major cause of variability between sources is the differences in stain processing and scanning. Hematoxylin stains the nuclei blue and eosin stains the cytoplasm and lamina propria pink,^14^ but in practice, raw image colour distributions may vary between WSIs, due to differences in stain composition, staining protocols, and colour responses of digital scanners.^16^ In other words, a method that performs well on one dataset from one laboratory on one scanner may not generalize to other datasets.

To combat these inherent variations in colour distributions and digitization artefacts, WSIs underwent stain processing prior to training. The variable way light is absorbed by the biopsies can be expressed with stain absorption vectors using Beer-Lambert’s law.^17^ These vectors can be estimated with the Macenko method,^18^ and used to process WSIs in 1 of 2 ways: stain normalization or stain jittering. Stain normalization is the process of changing the stain absorption vectors of a WSI, so that they match some fixed target stain absorption vectors without changing the stain concentrations. Stain normalizing the training data trains the neural network to make diagnoses on one specific (known) set of stain absorption vectors. Stain jittering is the process of perturbing the stain absorption vectors by a small random variable. Stain jittering the training data trains the neural network to make diagnoses independent of the stain absorption vectors.

In this study, we compared the effect of stain normalization and stain jittering on the training process of a state-of-the-art deep neural network as a tile-level classifier that was then used to give an overall WSI-level classifier. A deep neural network with no stain processing was trained as a base-line. Data from multiple sources were used for validation in order to test how well the classifier generalized on WSIs scanned with different scanners and from different laboratories.

We found that training on WSIs without any stain processing caused the neural network model to overfit on the training data, resulting in good performance on WSIs scanned on the same scanner and from the same laboratory, but poor performance on WSIs scanned on a different scanner or from a different laboratory. Additionally, we found that stain jittering the training data and stain normalizing the training data both improved the performance of the model on WSIs scanned on the same scanner and from the same laboratory as well as WSIs from other sources. When comparing models trained on stain jittered WSIs and models trained on stain normalized WSIs, stain jittering resulted in better performance on WSIs scanned on the same scanner and from the same laboratory, but stain normalizing resulted in better performance on WSIs scanned from a different scanner or from a different laboratory.

## Material and methods

### Dataset

The datasets used in this study are summarized in Table 1 and are composed of WSIs of H&E stained duodenal biopsies diagnosed by an expert pathologist. One dataset contained WSIs which were prepared in a laboratory which will be referred to as Centre 1, and scanned with a Ventana scanner. Labels for these scans were provided following a rigorous review of histology, serology, clinical details, and haemoglobin levels.^13^ This dataset was split into training and validation datasets with the ratio 80:20. These datasets will be referred to as the Ventana training and Ventana validation datasets, respectively. The Ventana training dataset contained 331 WSIs with 190 CD-positive and 141 CD-negative. The Ventana validation dataset contained 83 WSIs with 48 CD-positive and 35 CD-negative.

**Table 1:** Summary of the datasets involved.

Name	Size	Positive	Negative	Format	Institution	Magnification
Ventana (train)	331	190	141	.tif	Centre 1	×40
Ventana (val)	83	48	35	.tif	Centre 1	×40
Hamamatsu	83	48	35	.ndpi	Centre 1	×20
Aperio 1	83	48	35	.svs	Centre 1	×40
Aperio 2	75	38	37	.svs	Centre 2	×40

The slides scanned on the Ventana scanner to make the Ventana validation dataset were rescanned on a Hamamatsu scanner and an Aperio scanner. These datasets will be referred to as the Hamamatsu and Aperio 1 datasets, respectively.

Another dataset contained H&E stained duodenal biopsies prepared in a different laboratory which will be referred to as Centre 2, and was scanned on an Aperio scanner. Labels for these scans were provided by an expert gastrointestinal pathologist during routine diagnostic reporting. This dataset was used for validation and contained 38 CD-positive and 37 CD-negative WSIs. This dataset will be referred to as the Aperio 2 dataset.

All datasets presented here and their collection and quality control have been described previously in Denholm et al.^13^ (the Ventana and Aperio 2 datasets are referred to as H and A, respectively, in Denholm et al.^13^).

Validating on the Ventana validation dataset was used to assess how well a given model generalizes to WSIs of the same laboratory and scanner, while validating on the Hamamatsu and Aperio 1 datasets was used to assess how well a given model generalizes onto WSIs of the same laboratory and different scanner, and validating on the Aperio 2 dataset was used to assess how well a given model generalizes on WSIs of a different laboratory and scanner. Additionally, validation on the Aperio 1 and 2 datasets was used to compare the performance of a given model on WSIs from the same laboratory and from a different laboratory.

Examples of the WSIs in the Ventana, Hamamatsu, Aperio 1, and Aperio 2 datasets can be seen in Fig. 1.

### Ethical approval

Full ethical approval has been obtained for all anonymized biopsy, clinical, and patient data (IRAS: 162057; PI: Prof. E. Soilleux).

### Five-step pipeline for biopsy classification

#### Tile selection

Firstly, the WSIs were split into 224px×224px tiles at ×10 magnification as this resolution represented a balance between including essential contextual information (overall tissue morphology) and finer structure detail (e.g., preserving the ability to distinguish between epithelial and non-epithelial nuclei). These tiles were extracted using a sliding window. In inference/validation, the sliding window stride was half the tile length (112px). In training, a sliding window stride of a quarter the tile length (56px) was used instead. A smaller window stride in the training step was used to compensate the effect of rotational cropping (see the following section). The tissue was segmented using Otsu thresholding,^19^ and only tiles which contained at least 10% tissue were retained. The sliding window prevented tissue from the edges of the biopsy from being removed by this process.

#### Data augmentation

Data augmentation was applied to the tiles before each training step. In all experiments, random rotations and reflections were applied to the training data. This trained the models to make rotation- and reflection-independent diagnoses. Each tile was rotated by a random angle sampled from a $U_{\left[0^{{}^{\circ}},360^{{}^{\circ}}\right]}$ distribution before each training step. Since rotating an image by angles other than multiples of $90^{{}^{\circ}}$ resulted in data loss, the WSIs were split into tiles of size $\left\lfloor \sqrt{2}\times 224\mathrm{px}\right\rfloor =316\mathrm{px}$ and were cropped at the centre down to a tile of length 224px after rotation. To ensure the centre crop did not remove diagnostically relevant data during training, the stride of the sliding window was reduced by half (to 56px) in the training step. Each tile was reflected over the y-axis with a probability of 0.5 before each training step.

#### Optional application of stain normalization and jittering

To eradicate confounding effects of non-diagnostically relevant factors, such as variations in staining and use of different scanning equipment, tile-specific stain absorption vectors were estimated using the Macenko method.^18^ Where stain normalization was applied, the tiles were reconstructed using a fixed matrix of stain absorption vectors. Stain jittering is an alternative method to training a model to make stain variation-independent diagnoses. Instead of fixing the stain absorption vectors, they were randomly perturbed by multiplying each element of each vector by a $U_{\left[0.75,1.25\right]}$ random variable before each training step. Stain normalization reduces the size of the domain, whereas stain jittering increases the number of data points sampled from the domain.

Stain normalization was applied to tiles in the validation datasets *only* when stain normalization was applied to tiles in the training dataset. In all other cases, the stains of the tiles in the validation dataset were not processed. This was done because using stain normalized tiles trains the model to detect CD in tiles with some fixed stain absorption vectors, whereas using unprocessed tiles or stain jittered tiles trains the model to detect CD in tiles with stain absorption vectors present in the training data.

The code for stain normalization and jittering was thoroughly tested before experiments were conducted. Additionally, before training and validation in all experiments, a grid of 100 example tiles was saved as a .jpeg image for quality assurance. Examples of these grids of tiles are shown in Supplementary Fig. 1.

#### Tile-level classification

A ResNet-34 architecture,^15^ which was pretrained on the ImageNet dataset,^20^ was then trained on the Ventana training dataset to predict whether a given tile originated from a CD-positive or CD-negative biopsy. A binary cross-entropy function was used to calculate the loss. An Adam optimizer^21^ was used with learning rate $\lambda =10^{-4}$ and beta parameters $\beta =\left(0.9,0.999\right)$. The model was trained for 100 epochs with a batch size of 100 tiles. The performance of each model was measured on both a tile-level and a WSI-level. The following metrics were observed at tile-level:

1. **Tile Accuracy:** The percentage of tiles that were correctly classified by the model.
2. **Tile Sensitivity:** The percentage of tiles from a coeliac biopsy that were correctly labelled by the model.
3. **Tile Specificity:** The percentage of tiles from a negative biopsy that were correctly labelled by the model.
4. **Tile Precision:** The percentage of tiles predicted positive by the model that were correctly labelled.
5. **Tile F1 Score:**
  $$F1=2\times \frac{\;\mathrm{tile}\;\mathrm{sensitivity}\times \mathrm{tile}\;\mathrm{precision}\;}{\;\mathrm{tile}\;\mathrm{sensitivity}+\mathrm{tile}\;\mathrm{precision}\;}.$$

#### WSI-level classification

The WSI-level classification was made by thresholding over the mean of the tile-level predictions. The effect of the sensitivity and specificity with different thresholds was plotted as a ROC curve. A biopsy was labelled as positive if the ratio of positive tiles to total tiles exceeded some predictive threshold $\gamma \in \left[01\right]$. The threshold that maximizes the accuracy was calculated when validating on each dataset. The WSI-level accuracy, sensitivity, specificity, and F1 score were all calculated with this threshold. The following metrics were observed at WSI-level:

1. **WSI Threshold:** The percentage of tiles needed to be positive in order to classify the WSI as positive.
2. **WSI Accuracy:** The percentage of biopsies that were correctly labelled.
3. **WSI Sensitivity:** The percentage of positive biopsies that were correctly labelled.
4. **WSI Specificity:** The percentage of negative biopsies that were correctly labelled.
5. **WSI Precision:** The percentage of biopsies predicted positive by the model that were correctly labelled.
6. **WSI F1 Score:**
  $$F1=2\times \frac{\;\mathrm{biopsy}\;\mathrm{sensitivity}\times \mathrm{biopsy}\;\mathrm{precision}\;}{\;\mathrm{biopsy}\;\mathrm{sensitivity}+\mathrm{biopsy}\;\mathrm{precision}\;}.$$
7. **Receiver Operator Characteristic (ROC):** The plot of the true-positive rate (sensitivity) against the false-positive rate (1-specificity) at different thresholds.
8. **AUC:** The area under the ROC curve.

## Results

**Fig. 2:**
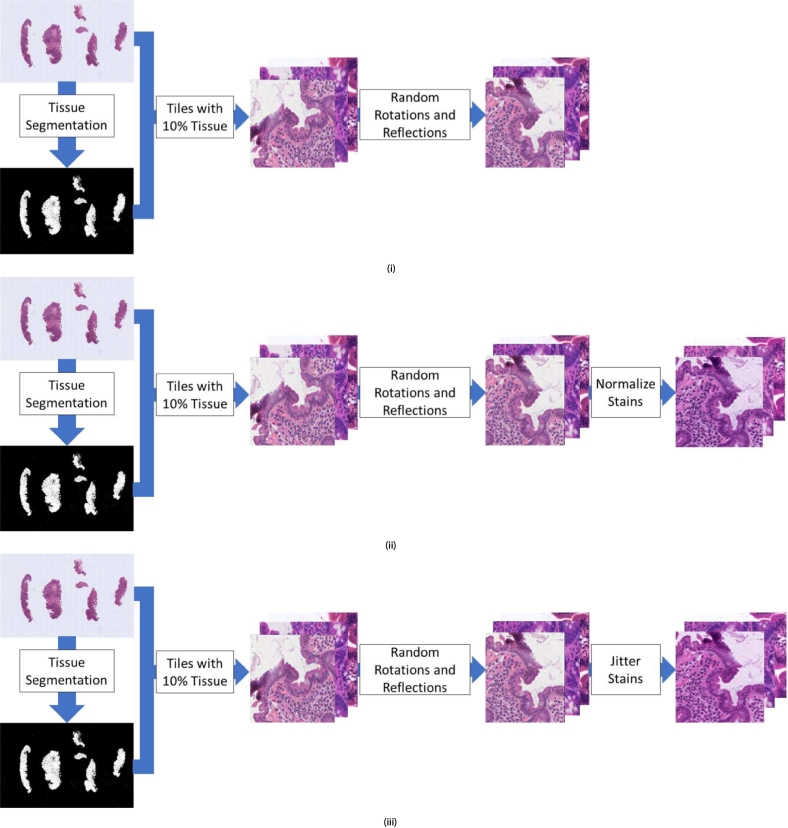
Tile selection and preprocessing steps: (i) Experiment 1 where the model is trained with no stain processing (NP model). (ii) Experiment 2 where the model trains on stain normalized data (SN model). (iii) Experiment 3 where the model trains on stain jittered data (SJ model). In all experiments, tissue was segmented using Otsu thresholding^19^ and only tiles for which at least 10% was tissue were included in training. These tiles were then randomly rotated and reflected as data augmentation.

In order to test the effect of stain normalization and jittering on CD diagnosis, 3 experiments were conducted. In each experiment, a neural network was trained on the Ventana training dataset as described in the methods section. In experiment 1 (baseline), the model was trained on tiles with no stain processing. In experiment 2, the stains of the tiles were normalized to the stain absorption vectors of a preselected target patch (from a dataset independent of this work) before training. In experiment 3, stain jittering was applied to the tiles before training by multiplying each element of each stain absorption vector by a $U_{\left[0.75,1.25\right]}$ random variable. These experiments are visualized in Fig. 2. The models trained on tiles with no stain processing, stain normalization, and stain jittering will be referred to as the NP, SN, and SJ models, respectively (see Fig. 2).

**Fig. 3:**
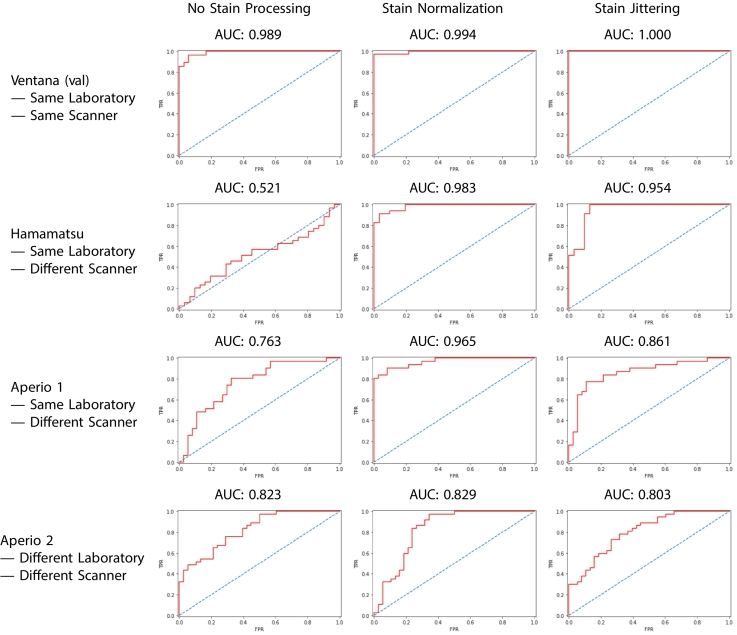
The WSI-level ROC curves of each model on each dataset. Left to right: The ROC curves of the models trained with no stain processing (NP model), trained with stain normalization (SN model), and trained with stain jittering (SJ model). Top to bottom: The ROC curves when validating on the Ventana dataset, Hamamatsu dataset, Aperio 1 dataset, and the Aperio 2 dataset.

To analyse the generalizability of the models, each model was validated on the Ventana validation, Hamamatsu, Aperio 1, and Aperio 2 datasets. A biopsy was labelled as positive if the ratio of positive tiles to total tiles exceeded some predictive threshold $\gamma \in \left[01\right]$. The result of different values for γ are shown in the ROC curves in Fig. 3. The threshold that maximized the accuracy was calculated when validating on each dataset. The WSI-level accuracy, sensitivity, specificity, and F1 score were all calculated with this threshold. This analysis summarizes how well the model separates positive and negative biopsies.

While the WSI accuracy, sensitivity, specificity, precision, F1 score, and ROC AUC (which will from now be referred to as the AUC) of each model on each dataset was recorded, the performance of each model on each dataset was assessed using the WSI F1 score and AUC. These metrics were picked over accuracy in order to counter-act the imbalance of positive and negative WSIs in each dataset, as well as the difference between the positive:negative ratio in the Aperio 2 dataset and the other datasets.

The AUCs for all 3 models on all 4 datasets are summarized in Table 2 and all other metrics and their 95% confidence intervals in Fig. 2. All other metrics can be found in the supplementary material.

**Table 2:** WSI-level accuracy, sensitivity, specificity, precision, and F1 scores with 95% confidence intervals for the 3 models on the Ventana, Hamamatsu, and Aperio datasets. **Bold** values signify the model that performed the best in the given metric and on the given dataset.

Dataset	Metrics	NP Model	SN Model	SJ Model
Ventana (val)	WSI Accuracy	0.953±0.012	0.984±0.007	1.000±0.000
	WSI Sensitivity	0.964±0.013	1.000±0.000	1.000±0.000
	WSI Specificity	0.944±0.018	0.972±0.012	1.000±0.000
	WSI Precision	0.931±0.018	0.966±0.013	1.000±0.000
	WSI F1 Score	0.947±0.012	0.982±0.007	1.000±0.000
Hamamatsu	WSI Accuracy	0.475±0.055	0.951±0.024	0.951±0.024
	WSI Sensitivity	1.000±0.000	0.962±0.021	0.885±0.045
	WSI Specificity	0.086±0.056	0.943±0.029	1.000±0.000
	WSI Precision	0.448±0.073	0.926±0.036	1.000±0.000
	WSI F1 Score	0.619±0.053	0.943±0.024	0.939±0.0261
Aperio 1	WSI Accuracy	0.742±0.048	0.909±0.031	0.833±0.040
	WSI Sensitivity	0.806±0.056	0.903±0.040	0.774±0.030
	WSI Specificity	0.686±0.078	0.914±0.049	0.886±0.025
	WSI Precision	0.694±0.066	0.903±0.040	0.857±0.025
	WSI F1 Score	0.746±0.048	0.903±0.033	0.814±0.043
Aperio 2	WSI Accuracy	0.663±0.055	0.846±0.041	0.693±0.054
	WSI Sensitivity	0.763±0.071	0.921±0.045	0.895±0.045
	WSI Specificity	0.541±0.084	0.784±0.066	0.486±0.085
	WSI Precision	0.673±0.078	0.780±0.068	0.642±0.079
	WSI F1 Score	0.727±0.051	0.847±0.041	0.747±0.050

On the Ventana validation dataset, the NP, SN, and SJ models correctly identified the presence of CD in the WSIs with 95.3%, 98.4%, and 100.0% accuracy, respectively, and F1 score of 0.947, 0.982, and 1.000, respectively. The AUCs of all models exceeded 99.0%. The SJ model correctly classified all biopsies in this dataset (i.e., using a validation set, the slides for which were performed in the same laboratory and scanned on the same scanner as the training set).

Despite the NP model demonstrating 95.3% accuracy on the Ventana dataset, its accuracy and F1 score on the Hamamatsu dataset were 47.5% and 0.619, respectively. The NP model classified almost all biopsies positive with sensitivity of 1.000 and specificity 0.086. Both the SN and SJ models correctly identified CD with accuracy 95.1%, but the F1 score of the SN model was higher than that of the SJ model with values of 0.943 and 0.939, respectively, indicating that the SN model outperforms the SJ model when the validation set comprises slides produced in the same laboratory but scanned on a different scanner. The difference of F1 scores between the 2 models is due to the differences in sensitivity and specificity. The SN model showed sensitivity 0.962 and specificity 0.943, whereas the SJ model showed a larger difference between these 2 metrics with sensitivity 0.885 and specificity 1.000.

On the Aperio 1 dataset, the SN model performed best in both accuracy and F1 score with accuracy 90.9% and F1 score 0.903. In comparison, the SJ model correctly classified CD with accuracy 83.3% and F1 score 0.814 and the NP model correctly classified CD with accuracy 74.2% and F1 score 0.746.

On the Aperio 2 dataset, the SN model again performed best in both accuracy and F1 score with accuracy 84.6% and F1 score 0.847. In comparison, the SJ model correctly classified CD with accuracy 69.3% and F1 score 0.747 and the NP model correctly classified CD with accuracy 66.3% and F1 score 0.727. These results indicate that the SN model outperforms the SJ model when the validation set comprises slides produced in a different laboratory and/or scanned on a different scanner.

## Discussion

After training on the Ventana training dataset, the NP model (trained on data with no stain processing), SN model (trained on data with stain normalization), and SJ model (trained on data with stain jittering) performed well on the Ventana validation dataset (slides produced in the same laboratory and scanned with the same scanner). All models achieved accuracy above 95.0%, F1 scores above 0.94 and ROC AUCs above 0.98. The SJ model in particular correctly labelled all WSIs in this dataset. This suggests that using stain jittering improves the performance of the neural network on data of the same source as the training data. This is possibly due to the increased diversity of training data in the SJ model. Both the SN and SJ models performed better than the NP model in accuracy, F1 score and AUC. These results confirm that there is stain variation between WSIs from the same scanner and laboratory, and illustrate the importance of stain processing.

While the NP model performed well on the Ventana validation dataset, the accuracy, F1 score, and AUC decreased dramatically when validating on the Hamamatsu dataset. The sensitivity and specificity of this model was 1.000 and 0.086 on this dataset, respectively. In other words, the NP model labelled the vast majority of the WSIs positive. This is an interesting observation, because the biopsies in the Hamamatsu dataset are identical to those in the Ventana dataset, with the only difference being the scanner used. Our results demonstrate that a change in apparatus can significantly impair performance without proper stain processing.

Unlike the NP model, both the SN and SJ models showed only a slight drop in performance between the Ventana and Hamamatsu datasets. Both models achieved the same accuracy of 95.1% accuracy. However, the SN model slightly outperformed the SJ model in F1 score with scores of 0.943 and 0.939, respectively. This is to be expected as in theory, the SN model should perform identically on the Ventana and Hamamatsu datasets as the purpose of stain normalization is to remove the effect of external variables such as scanning equipment and focus entirely on the features of the biopsy. On the other hand, the SJ model is based on the principle that the diagnosis should be independent of the stain absorption vectors with stain jittering permitting a more complex model. Thus, when classifying the same biopsies with changes to the colour distribution, the SN model should perform better. The slight performance decrease of the SN model between the Ventana and Hamamatsu datasets could be attributed to non-colour scanner-related artefacts such as inconsistent microns per pixel and small out-of-focus areas.

On the Aperio 1 dataset, the SN model performed better than the SJ model in both accuracy and F1 score, showing the importance of stain normalization for generalizing models across multiple datasets. On the Aperio 1 dataset, the SN and SJ models had accuracy of 90.9% and 83.3%, respectively, and F1 scores of 0.903 and 0.815, respectively. This provides further evidence that stain normalization improves generalizability between different scanners more effectively than stain jittering.

The accuracy and F1 scores of both the SN and SJ models were lower when validating on the Aperio 1 dataset than when validating on the Hamamatsu dataset; their accuracies decreased by 0.041% and 0.118%, respectively, and their F1 scores decreased by 0.040 and 0.125, respectively. The Aperio 1 dataset contains WSIs of the same slides as those in the Ventana validation and Hamamatsu datasets, so this decrease in performance is unexpected as a biopsy scanned on 2 different scanners should theoretically result in identical WSIs after stain normalization. Again, this is likely caused by scanner-related artefacts. It is important to note that the decreases in performance when changing scanners is smaller in the SN model than in the SJ model. This provides evidence that stain normalizing the training data improves the generalizability of the model between scanners more than stain jittering the training data.

Interestingly, the NP model performed much better on the Aperio 1 dataset than on the Hamamatsu dataset. The NP model achieved an accuracy, F1 score and AUC of 74.2%, 0.746 and 0.763, respectively, on the Aperio 1 dataset. This suggests that a model trained on Ventana WSIs generalizes to Aperio WSIs better than Hamamatsu WSIs, and supports the hypothesis that models trained on WSIs that have not undergone stain normalization or jittering are less reliable when validating on WSIs scanned on a different scanner to the training data.

The WSIs in the Aperio 2 dataset contain biopsies unseen in all other previously mentioned datasets. Additionally, these biopsies were prepared in a different laboratory to the biopsies in the other datasets. Theoretically, the WSIs in this dataset should have similar stain absorption vectors to those in the Aperio 1 dataset as they were all scanned with the same Aperio scanner. The change in laboratory then explains why the performance of all 3 models decreased on this dataset. While the AUC of all models were fairly similar (0.823, 0.829, and 0.803 for the NP, SN, and SJ models, respectively), the SN model performed best in accuracy and F1 score at 84.6% and 0.847, respectively. This greatly outperformed the SJ model with accuracy and F1 score of 69.3% and 0.747, respectively. This suggests that standard normalization, unlike stain jittering, improves generalizability between different laboratories as well as different scanners.

Interestingly, while the accuracy of the NP model on the Aperio 2 dataset was lower than that on the Aperio 1 dataset, the F1 scores were similar for both Aperio datasets—especially when compared to the difference in performance of the NP model on the Ventana and Hamamatsu datasets. The NP model had an accuracy and F1 score of 66.3% and 0.727 on Aperio 2 dataset, respectively. This suggests that the performance of the NP model on the Aperio datasets is directly related to the stain absorption vectors of the biopsies and less on characteristics of the biopsy images that were a consequence of which laboratory processed them.

There are several ways these experiments could be improved. For example, the model could be trained on a larger dataset. The training data was not expanded here because it was essential for this study to ensure that all training data came from the same source. However, the small dataset may have affected the performance of the models. For example, it is expected that stain normalization performs better than stain jittering when trained on smaller datasets, because the space of stain normalized biopsies is strictly smaller than that of non-processed biopsies; a model training on the space of stain normalized biopsies learns to classify on a smaller domain.

Another challenge to be discussed in future work is the potential for ground-truth noise. The biopsies in this study were labelled by expert pathologists. Diagnosing CD through the analysis of duodenal biopsies is a difficult and subjective task, and neither the binary positive/negative labels nor the models used in this study account for potential uncertainty or mislabelling.^9^^,^^10^ In future work, semi-supervised learning will be used to mitigate this. Semi-supervised learning allows the neural network to label unlabelled data and relabel outlier data. It has been used successfully in tasks with ground-truth noise.^22^, ^23^, ^24^

This study shows the importance of stain processing when training neural network-based models on WSI biopsy data. We show that when training such models, stain jittering improves the performance of the model on data from the same source as the training data, while stain normalization improves the performance of the model when validating on data processed in different laboratories and/or scanned with different scanners, making the model more generalizable and reliable.

## Author Statement

The code for processing and analysing the WSI data was written by B.A.S and J.D with equal contribution.

The project was initialized by E.J.S and supervised by E.J.S and C.B.S.

All authors were given the opportunity to review and comment on the manuscript.

## Funding

This work was supported by the Pathological Society of Great Britain and Northern Ireland and 10.13039/100004330GlaxoSmithKline.

## Appendix A Supplementary data

Supplementary material Image 1
