# Supplementary material for: Stain normalization gives greater generalizability than stain jittering in neural network training for the classification of coeliac disease in duodenal biopsy whole slide images
Source: J Pathol Inform. 2023 Jul 19;14:100324. doi: 10.1016/j.jpi.2023.100324 (PMC10416012; doi:10.1016/j.jpi.2023.100324)
Supplement: Supplementary file 1 — Supplementary material [file mmc1.docx]

## Supplementary Material


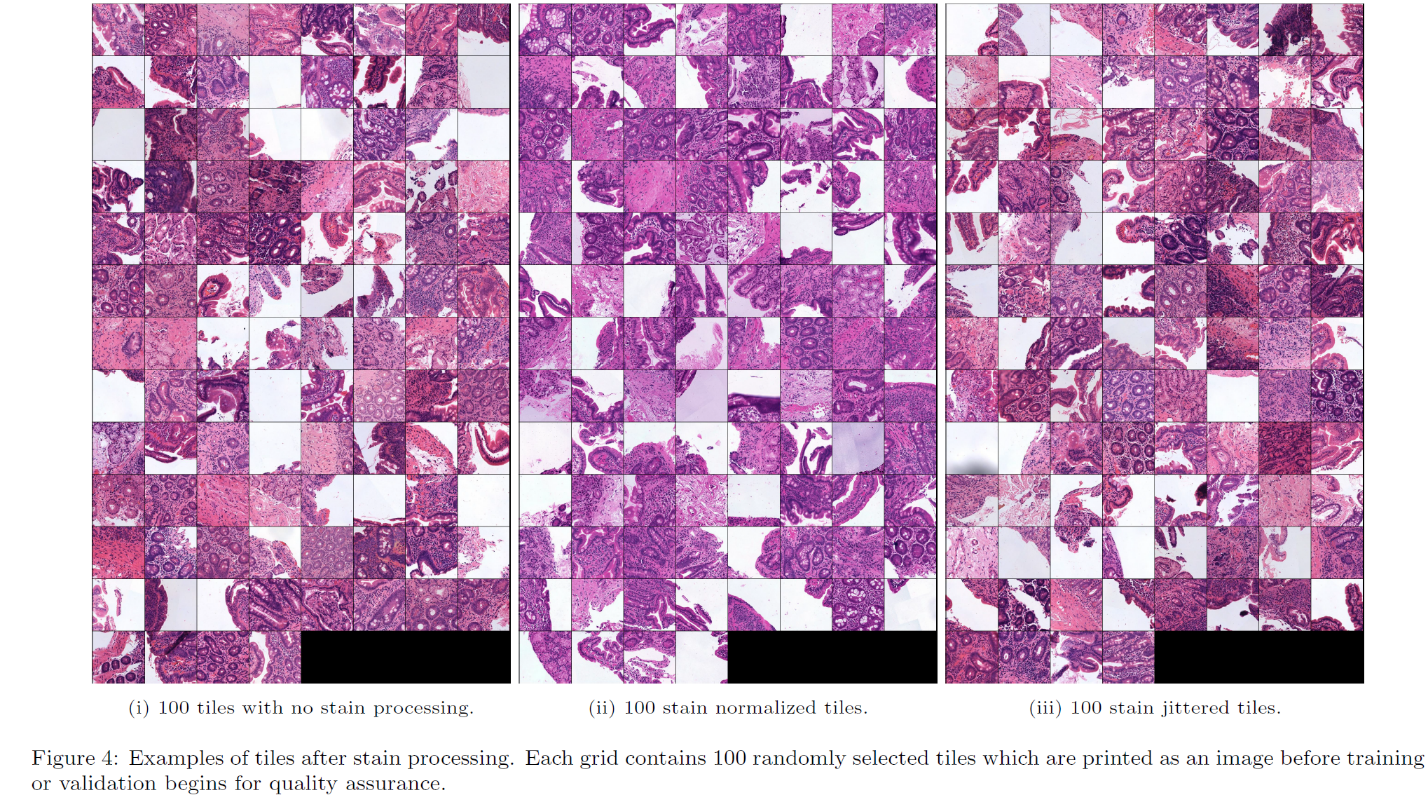


Figure 1: Examples of tiles after stain processing. Each grid contains 100 randomly selected tiles which are printed as an image before training or validation begins for quality assurance.

| Metrics | NP Model | SN Model | SJ Model |
| --- | --- | --- | --- |
| Tile Accuracy | 0.898 | 0.912 | 0.821 |
| Tile Sensitivity | 0.894 | 0.891 | 0.758 |
| Tile Specificity | 0.902 | 0.932 | 0.877 |
| Tile Precision | 0.890 | 0.921 | 0.846 |
| Tile F1 Score | 0.892 | 0.905 | 0.799 |
| WSI Threshold | 0.472 | 0.476 | 0.457 |
| WSI Accuracy | 0.953 | 0.984 | 1.000 |
| WSI Sensitivity | 0.964 | 1.000 | 1.000 |
| WSI Specificity | 0.944 | 0.972 | 1.000 |
| WSI Precision | 0.931 | 0.966 | 1.000 |
| WSI F1 Score | 0.947 | 0.982 | 1.000 |

Table 3: WSI and tile metrics for the three models on the validation set from the Ventana dataset.

| Metrics | NP Model | SN Model | SJ Model |
| --- | --- | --- | --- |
| Tile Accuracy | 0.454 | 0.672 | 0.691 |
| Tile Sensitivity | 0.998 | 0.953 | 0.890 |
| Tile Specificity | 0.004 | 0.439 | 0.527 |
| Tile Precision | 0.454 | 0.584 | 0.609 |
| Tile F1 Score | 0.624 | 0.725 | 0.723 |
| WSI Threshold | 0.979 | 0.828 | 0.840 |
| WSI Accuracy | 0.475 | 0.951 | 0.951 |
| WSI Sensitivity | 1.000 | 0.962 | 0.885 |
| WSI Specificity | 0.086 | 0.943 | 1.000 |
| WSI Precision | 0.448 | 0.926 | 1.000 |
| WSI F1 Score | 0.619 | 0.943 | 0.939 |

Table 4: WSI and tile metrics for the three models on the validation set from the Hamamatsu dataset.

| Metrics | NP Model | SN Model | SJ Model |
| --- | --- | --- | --- |
| Tile Accuracy | 0.686 | 0.715 | 0.659 |
| Tile Sensitivity | 0.748 | 0.851 | 0.555 |
| Tile Specificity | 0.623 | 0.582 | 0.762 |
| Tile Precision | 0.663 | 0.668 | 0.697 |
| Tile F1 Score | 0.703 | 0.748 | 0.618 |
| WSI Threshold | 0.421 | 0.668 | 0.397 |
| WSI Accuracy | 0.742 | 0.909 | 0.833 |
| WSI Sensitivity | 0.806 | 0.903 | 0.774 |
| WSI Specificity | 0.686 | 0.914 | 0.886 |
| WSI Precision | 0.694 | 0.903 | 0.857 |
| WSI F1 Score | 0.746 | 0.903 | 0.814 |

Table 5: WSI and tile metrics for the three models on the validation set of the Aperio 1 dataset.

| Metrics | NP Model | SN Model | SJ Model |
| --- | --- | --- | --- |
| Tile Accuracy | 0.659 | 0.644 | 0.628 |
| Tile Sensitivity | 0.846 | 0.936 | 0.736 |
| Tile Specificity | 0.407 | 0.252 | 0.481 |
| Tile Precision | 0.657 | 0.627 | 0.656 |
| Tile F1 Score | 0.740 | 0.751 | 0.618 |
| WSI Threshold | 0.619 | 0.859 | 0.534 |
| WSI Accuracy | 0.663 | 0.846 | 0.693 |
| WSI Sensitivity | 0.763 | 0.921 | 0.895 |
| WSI Specificity | 0.541 | 0.784 | 0.486 |
| WSI Precision | 0.673 | 0.780 | 0.642 |
| WSI F1 Score | 0.727 | 0.847 | 0.747 |

Table 6: WSI and tile metrics for the three models on the validation set of the Aperio 2 dataset.
